# Supplementary material for: Deterministic realization of collective measurements via photonic quantum walks
Source: Nat Commun. 2018 Apr 12;9:1414. doi: 10.1038/s41467-018-03849-x (PMC5897416; doi:10.1038/s41467-018-03849-x)
Supplement: Supplementary file 1 — Supplementary Information [file 41467_2018_3849_MOESM1_ESM.pdf]

Supplementary Information: Deterministic realization of collective measurements via photonic quantum walks

Z. Hou et al.

Supplementary Note 1: Realization of the collective SIC-POVM via quantum walks

Recently, quantum walks were proposed as a recipe for implementing general POVMs on a single qubit [1], which have been demonstrated in experiments [2, 3]. In a one-dimensional discrete quantum walk, the system state is characterized by two degrees of freedom $|x, c\rangle$, where $x = \cdots, -1, 0, 1, \cdots$ denotes the walker position, and $c = 0, 1$ represents the coin state. The dynamics of each step is described by a unitary transformation of the form $U(t) = TC(t)$, where T is the conditional translation operator

$$T = \sum_x |x+1, 0\rangle\langle x, 0| + |x-1, 1\rangle\langle x, 1|, \tag{1}$$

and $C(t) = \sum_x |x\rangle\langle x| \otimes C(x,t)$ with $C(x,t)$ being site-dependent coin operators. A general POVM on a qubit can be realized by engineering the coin operators $C(x,t)$ followed by measuring the walker position after certain steps. However, little is known in the literature on realizing POVMs on higher dimensional systems based on quantum walks. Here we offer a recipe to extending the capabilities of quantum walks.

For concreteness, we focus on the collective SIC-POVM on a two-qubit system, which is composed of five outcomes,

$$E_j = \frac{3}{4}(|\psi_j\rangle\langle\psi_j|)^{\otimes 2}, \quad E_5 = |\Psi_-\rangle\langle\Psi_-|, \tag{2}$$

where $|\Psi_-\rangle = \frac{1}{\sqrt{2}}(|\overline{01}\rangle - |\overline{10}\rangle)$ is the singlet, and

$$\begin{aligned} |\psi_1\rangle =& |\overline{0}\rangle, & |\psi_2\rangle =& \frac{1}{\sqrt{3}}(|\overline{0}\rangle + \sqrt{2}|\overline{1}\rangle), \\ |\psi_3\rangle =& \frac{1}{\sqrt{3}}(|\overline{0}\rangle + \mathrm{e}^{\frac{2\pi}{3}\mathrm{i}}\sqrt{2}|\overline{1}\rangle), & |\psi_4\rangle =& \frac{1}{\sqrt{3}}(|\overline{0}\rangle + \mathrm{e}^{-\frac{2\pi}{3}\mathrm{i}}\sqrt{2}|\overline{1}\rangle) \end{aligned} \tag{3}$$

form a symmetric informationally complete POVM (SIC-POVM) on a qubit [4, 5]. Here the "overline" on $\overline{0}, \overline{1}$ is added to distinguish logical quantum states from physical quantum states of the walker and the coin. The Bloch vectors of the four states $|\psi_j\rangle$ for $j = 1, 2, 3, 4$ are given by $\mathbf{r}_1 = (0, 0, 1)$, $\mathbf{r}_2 = (\frac{2\sqrt{2}}{3}, 0, -\frac{1}{3})$, $\mathbf{r}_3 = (-\frac{\sqrt{2}}{3}, \frac{\sqrt{6}}{3}, -\frac{1}{3})$ and $\mathbf{r}_4 = (-\frac{\sqrt{2}}{3}, -\frac{\sqrt{6}}{3}, -\frac{1}{3})$, which form a regular tetrahedron inside the Bloch sphere.

To realize the collective SIC-POVM using quantum walks, the coin qubit and the walker in positions 1 and -1 are taken as the two-qubit system of interest, while the other positions of the walker act as an ancilla. With this choice, the collective SIC-POVM can be realized via five-step quantum walks as illustrated in Figure 1d in the main text, with nontrivial coin operators given by

$$\begin{aligned} C(-1,1) =& \frac{1}{\sqrt{3}}\begin{pmatrix} 1 & \sqrt{2} \\ \sqrt{2} & -1 \end{pmatrix}, & C(-2,2) =& \begin{pmatrix} 0 & 1 \\ 1 & 0 \end{pmatrix}, & C(0,2) =& \frac{1}{2}\begin{pmatrix} \sqrt{3} & 1 \\ 1 & -\sqrt{3} \end{pmatrix}, \\ C(1,3) =& \frac{1}{\sqrt{3}}\begin{pmatrix} \sqrt{2} & 1 \\ 1 & -\sqrt{2} \end{pmatrix}, & C(0,4) =& \begin{pmatrix} 1 & 0 \\ 0 & -1 \end{pmatrix}, & C(-1,5) =& \frac{1}{2}\begin{pmatrix} 1-\mathrm{i} & 1+\mathrm{i} \\ -1+\mathrm{i} & 1+\mathrm{i} \end{pmatrix}, \\ C(2,2) =& C(0,2), & C(-1,3) =& C(-1,1), & C(-2,4) =& C(-2,2). \end{aligned} \tag{4}$$

To see this, note that a general logical two-qubit pure state

$$|\Psi_0\rangle = a|\overline{00}\rangle + b|\overline{01}\rangle + c|\overline{10}\rangle + d|\overline{11}\rangle, \quad |a|^2 + |b|^2 + |c|^2 + |d|^2 = 1 \tag{5}$$

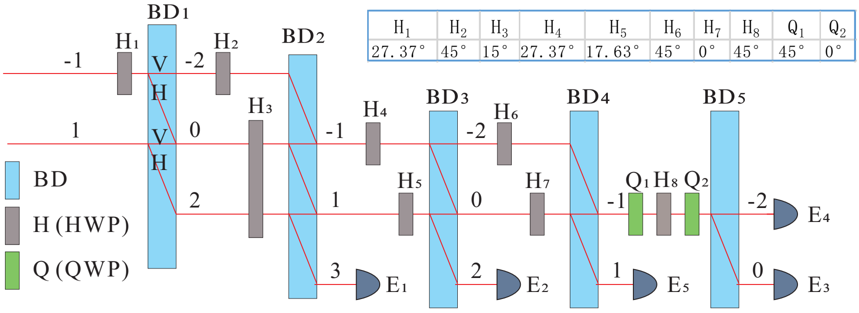

Supplementary Figure 1: Realization of the collective SIC-POVM using five-step photonic quantum walks (cf. Figure 1d and Figure 2 in the main text). The polarization-encoded coin qubit and the walker in positions 1 and -1 are taken as the two-qubit system of interest, while the other positions of the walker act as an ancilla. Beam displacers (BDs) are used to realize the conditional translation operator T. Combinations of half wave plates (HWPs) and quarter wave plates (QWPs) are used to realize site-dependent coin operators $C(x,t)$. Five single-photon detectors (SPDs) E_1 to E_5 correspond to the five outcomes. The collective SIC-POVM is realized by choosing the rotation angles of the HWPs and QWPs according to the table embedded in the figure (identical to the table embedded in Figure 2 in the main text).

can be encoded into the initial state (corresponding to step $t=0$) of the walker-coin system as

$$|\Psi_0\rangle = a|1,0\rangle + b|1,1\rangle + c|-1,0\rangle + d|-1,1\rangle. \tag{6}$$

After step 1, the state $|\Psi_0\rangle$ evolves into

$$|\Psi_1\rangle = TC(t=1)|\Psi_0\rangle = a|2,0\rangle + b|0,1\rangle + \left(\sqrt{\frac{1}{3}}\,c + \sqrt{\frac{2}{3}}\,d\right)|0,0\rangle + \left(\sqrt{\frac{2}{3}}\,c - \sqrt{\frac{1}{3}}\,d\right)|-2,1\rangle. \tag{7}$$

Following a similar procedure, the state after step 5 reads

$$\begin{aligned}|\Psi_5\rangle =& \frac{\sqrt{3}}{2}a|6,0\rangle + \frac{\sqrt{3}}{6}(a+\sqrt{2}b+\sqrt{2}c+2d)|4,0\rangle + \frac{\sqrt{2}}{2}(-b+c)|2,0\rangle \\ &+ \frac{\sqrt{3}}{6}\mathrm{e}^{\frac{\pi}{4}\mathrm{i}}\big(a+\sqrt{2}\mathrm{e}^{-\frac{2\pi}{3}\mathrm{i}}b+\sqrt{2}\mathrm{e}^{-\frac{2\pi}{3}\mathrm{i}}c+2\mathrm{e}^{-\frac{4\pi}{3}\mathrm{i}}d\big)|0,0\rangle \\ &+ \frac{\sqrt{3}}{6}\mathrm{e}^{\frac{\pi}{4}\mathrm{i}}\big(a+\sqrt{2}\mathrm{e}^{\frac{2\pi}{3}\mathrm{i}}b+\sqrt{2}\mathrm{e}^{\frac{2\pi}{3}\mathrm{i}}c+2\mathrm{e}^{\frac{4\pi}{3}\mathrm{i}}d\big)|-2,1\rangle.\end{aligned} \tag{8}$$

Now measuring the position of the walker realizes the collective SIC-POVM as desired. To verify this claim, note that the probabilities of detecting the walker at positions $6,4,2,0,-2$ are respectively given by

$$\begin{aligned}\tilde{p}_6 =& \frac{3}{4}|a|^2 = \frac{3}{4}|\langle\psi_1\psi_1|\Psi_0\rangle|^2 = \langle\Psi_0|E_1|\Psi_0\rangle, \\ \tilde{p}_4 =& \frac{1}{12}|a+\sqrt{2}b+\sqrt{2}c+2d|^2 = \frac{3}{4}|\langle\psi_2\psi_2|\Psi_0\rangle|^2 = \langle\Psi_0|E_2|\Psi_0\rangle, \\ \tilde{p}_2 =& \frac{1}{2}|-b+c|^2 = |\langle\Psi_-|\Psi_0\rangle|^2 = \langle\Psi_0|E_5|\Psi_0\rangle, \\ \tilde{p}_0 =& \frac{1}{12}\big|a+\sqrt{2}\mathrm{e}^{-\frac{2\pi}{3}\mathrm{i}}b+\sqrt{2}\mathrm{e}^{-\frac{2\pi}{3}\mathrm{i}}c+2\mathrm{e}^{-\frac{4\pi}{3}\mathrm{i}}d\big|^2 = \frac{3}{4}|\langle\psi_3\psi_3|\Psi_0\rangle|^2 = \langle\Psi_0|E_3|\Psi_0\rangle, \\ \tilde{p}_{-2} =& \frac{1}{12}\big|a+\sqrt{2}\mathrm{e}^{\frac{2\pi}{3}\mathrm{i}}b+\sqrt{2}\mathrm{e}^{\frac{2\pi}{3}\mathrm{i}}c+2\mathrm{e}^{\frac{4\pi}{3}\mathrm{i}}d\big|^2 = \frac{3}{4}|\langle\psi_4\psi_4|\Psi_0\rangle|^2 = \langle\Psi_0|E_4|\Psi_0\rangle,\end{aligned} \tag{9}$$

So the detectors at the five positions $6,4,2,0,-2$ correspond to the five POVM elements E_1, E_2, E_5, E_3, E_4 specified in Eqs. (2) and (3). Note that a detector at position 6 after step 5 is equivalent to a detector at position 3 after step 2. Similarly, the detector at position 4 (position 2) after step 5 can be placed at position 2 (position 1) after step 3 (step 4) without changing the detection probability. This fact can be utilized to simplify the experimental design, as reflected in Figure 1 in the main text.

The above proposal can be realized using photonic quantum walks as illustrated in Supplementary Figure **1** as well as Figure 2 in the main text. In this scheme, the conditional translation operator is realized by beam displacers (BDs), which displace the H-component away from the V-component. The coin operators are realized by suitable combinations of half wave plates (HWPs) and quarter wave plates (QWPs) with rotation angles specified in Supplementary Figure **1**. Note that a HWP with rotation angle h and a QWP with rotation angle q realize the following unitary transformations

$$U_{\mathrm{H}}(h) = \begin{pmatrix} \cos 2h & \sin 2h \\ \sin 2h & -\cos 2h \end{pmatrix}, \quad U_{\mathrm{Q}}(q) = \frac{1}{\sqrt{2}} \mathrm{e}^{\frac{\pi}{4}\mathrm{i}} \begin{pmatrix} 1 - \mathrm{i}\cos 2q & -\mathrm{i}\sin 2q \\ -\mathrm{i}\sin 2q & 1 + \mathrm{i}\cos 2q \end{pmatrix}. \tag{10}$$

Based on this equation, it is straightforward to verify that the site-dependent coin operators presented in Eq. (4) are realized by the wave plates shown in Supplementary Figure **1** with rotation angles as specified.

Supplementary Note 2: State preparation

In this section we provide additional details on the preparation of walker-coin two-qubit states considered in the main text. Our discussion is based on the state preparation module illustrated in Figure 2 in the main text. Note that the quartz crystal and the HWP corresponding to α_2 are used only in the preparation of mixed states. In general, a walker-coin two-qubit state can be prepared by properly choosing the rotation angles α_1,α_2, h_1, h_2, h_3, q_1 and q_2 of the HWPs and QWPs shown in this module. The parameter choices for various states considered in the main text are specified in Supplementary Table **1**. Here $|\pm_z\rangle$, $|\pm_x\rangle$ and $|\pm_y\rangle$ denote the two eigenstates with eigenvalues ± 1 of σ_z, σ_x and σ_y, respectively, with Bloch vectors given by $(0,0,\pm1)$, $(\pm1,0,0)$ and $(0,\pm1,0)$; these states are used in the measurement tomography of the collective SIC-POVM. $\hat{E}_j$ for $j=1$ to 5 denote the five normalized POVM elements of the collective SIC-POVM (note that $\hat{E}_1 = |+_z+_z\rangle\langle+_z+_z|$); these states are used in the experimental verification of the collective SIC-POVM. The rest states in the table are studied in quantum state tomography with the collective SIC-POVM. To be specific, $|\psi(\theta)\rangle = \sin\theta|0\rangle + \cos\theta|1\rangle$ is a pure state parametrized by θ; $\frac{1}{\sqrt{2}}(1,0,1)$ and $\frac{1}{\sqrt{3}}(1,1,1)$ denote two pure states with Bloch vectors as specified; $\mathbf{s}_1$ and $\mathbf{s}_2$ denote quantum states whose Bloch vectors align with $\hat{\mathbf{s}}_1 = (0,0,-1)$ and $\hat{\mathbf{s}}_2 = (0.490, -0.631, 0.602)$, and with lengths s_1 and s_2, respectively.

In the preparation of $\hat{E}_5$, which is maximally entangled, the QWP corresponding to q_2 is removed, and h_3 is set at 0°. For all other states considered in this work, which are product states, this QWP is present, and h_3 is set at 45°. In the preparation of a product state, α_1, h_1, q_1 are used to control the state of the first qubit (walker), while α_2 (together with the quartz crystal), h_2, q_2 are used to control the state of the second qubit (coin). Specifically, the length of the Bloch-vector of the first qubit is determined by α_1, while the direction of the Bloch-vector is determined by h_1, q_1. In the preparation of the second qubit, α_2, h_2, q_2 play similar roles to α_1, h_1, q_1 for the first qubit. The parameters shown in Supplementary Table **1** apply to the preparation of a two-copy state, of which the walker qubit and the coin qubit are identical. Since the preparation of the two qubit states are independent, product states with different marginals can also be prepared with straightforward modification. For example, the product state $|+_z+_x\rangle$ can be prepared by choosing the following parameters:

$$\alpha_1 = q_1 = 0, \quad h_1 = h_3 = q_2 = 45°, \quad h_2 = -22.5°. \tag{11}$$

Supplementary Note 3: Quantum measurement tomography of the collective SIC-POVM

In this section, we provide more details on the measurement tomography of the collective SIC-POVM realized using photonic quantum walks. To perform measurement tomography, 36 states, the tensor products of the six eigenstates of three Pauli operators, were prepared according to the method described in the previous section and sent to the collective-measurement module. To reduce statistical fluctuation, each state was prepared and measured 35000 times. Then the five POVM elements were estimated from the measurement statistics using the maximum likelihood (ML) method developed in Ref. [6]. The five reconstructed POVM elements are shown in Supplementary Figure **2** in comparison with the ideal counterparts. The fidelities of the five POVM elements are 0.9991 ± 0.0001, 0.9979 ± 0.0007, 0.9870 ± 0.0008, 0.9927 ± 0.0002 and 0.9961 ± 0.0002, respectively; the overall fidelity of the POVM is 0.9946 ± 0.0002 (cf. the Methods section). These results show that the collective SIC-POVM was realized with very high quality.

Supplementary Table 1: Parameter choices in walker-coin two-qubit state preparation. Here $\alpha_1, \alpha_2, h_1, h_2, q_1, q_2$ are the rotation angles of the HWPs and QWPs shown in Figure 2 in the main text, and "quartz" denotes the quartz crystal in the same figure. $|\pm_z\rangle$, $|\pm_x\rangle$ and $|\pm_y\rangle$ denote the two eigenstates with eigenvalues ± 1 of σ_z, σ_x and σ_y; the parameters in the parentheses apply to the states with eigenvalue -1. $\hat{E}_j$ for $j = 1$ to 5 denote the five normalized POVM elements of the collective SIC-POVM (note that $\hat{E}_1 = |+_z +_z\rangle\langle +_z +_z|$). $|\psi(\theta)\rangle = \sin\theta|0\rangle + \cos\theta|1\rangle$ is a pure state. $\frac{1}{\sqrt{2}}(1,0,1)$ and $\frac{1}{\sqrt{3}}(1,1,1)$ denote two pure states with Bloch vectors as specified. $\mathbf{s}_1$ and $\mathbf{s}_2$ denote quantum states whose Bloch vectors align with $\hat{\mathbf{s}}_1 = (0,0,-1)$ and $\hat{\mathbf{s}}_2 = (0.490, -0.631, 0.602)$, and with lengths s_1 and s_2, respectively; $\alpha(s) = \frac{1}{4}\arccos(s)$. The quartz and the HWP corresponding to α_2 are used only in the preparation of mixed states (in the last two columns of the table). In the preparation of $\hat{E}_5$, the QWP corresponding to q_2 is removed.

States	$\|\pm_z\rangle$	$\|\pm_x\rangle$	$\|\pm_y\rangle$	$\hat{E}_2$	$\hat{E}_3$	$\hat{E}_4$	$\hat{E}_5$	$\frac{1}{\sqrt{2}}(1,0,1)$	$\frac{1}{\sqrt{3}}(1,1,1)$	$\|\psi(\theta)\rangle$	$\mathbf{s}_1$	$\mathbf{s}_2$
α_1	0	0	0	0	0	0	0	0	0	0	$\alpha(s_1)$	$\alpha(s_2)$
$h_1(^\circ)$	45(0)	-22.5(22.5)	-22.5(22.5)	-17.63	0	27.37	22.5	56.25	-24.95	$90-\frac{\theta}{2}$	0	45
$q_1(^\circ)$	0	45	0	-35.26	27.37	27.37	45	22.5	22.5	$-\theta$	0	19.57
$h_3(^\circ)$	45	45	45	45	45	45	0	45	45	45	45	45
$\alpha_2(^\circ)$	–	–	–	–	–	–	–	–	–	–	$\alpha(s_1)$	$\alpha(s_2)$
quartz	–	–	–	–	–	–	–	–	–	–	0	0
$h_2(^\circ)$	45(0)	-22.5(22.5)	-22.5(22.5)	-17.63	0	27.37	45	56.25	-24.95	$90-\frac{\theta}{2}$	0	45
$q_2(^\circ)$	0	45	0	-35.26	27.37	27.37	–	22.5	22.5	$-\theta$	0	19.57

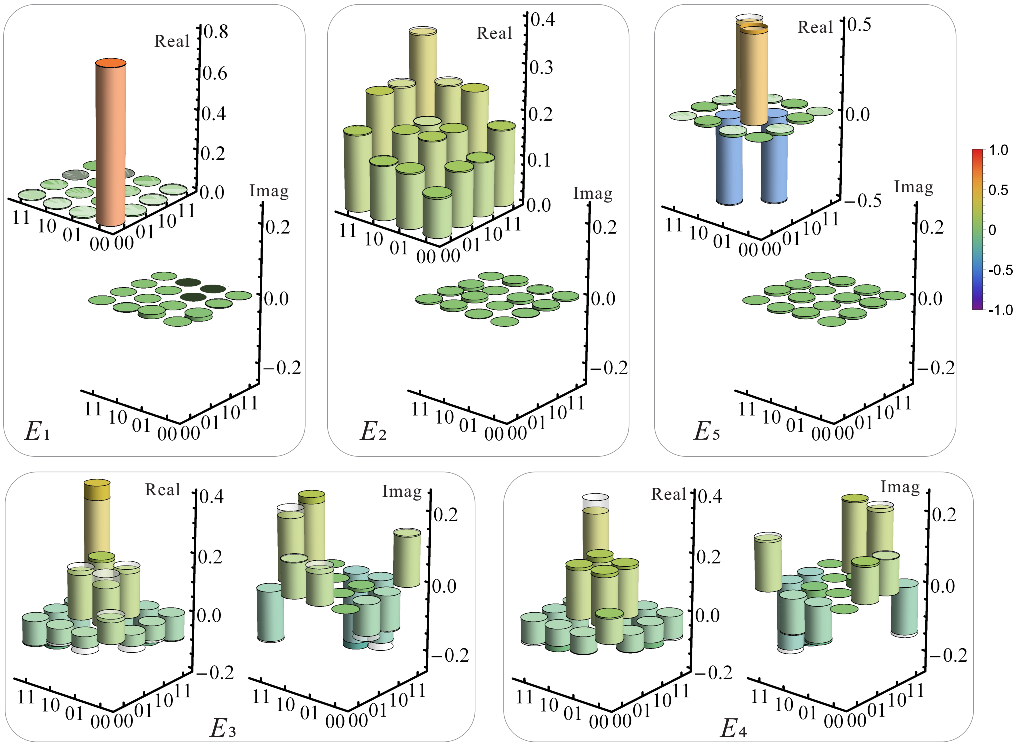

Supplementary Figure 2: Results on measurement tomography of the collective SIC-POVM realized in the experiment. The matrix elements of the real (Real) and imaginary (Imag) parts of the five POVM elements E_1 to E_5 are plotted using solid colours. For comparison, the counterparts of the ideal POVM are plotted as wire frames.

Supplementary Note 4: Scaling of the mean infidelity with the sample size

In this supplement, we provide additional details on the scaling of the mean infidelity with the sample size in quantum state tomography with the collective SIC-POVM. To complement the results presented in the main text, we first illustrate the scaling of the mean infidelity $1-F$ achieved by the collective SIC-POVM for mixed states. We then present the scaling exponents for both pure states and mixed states.

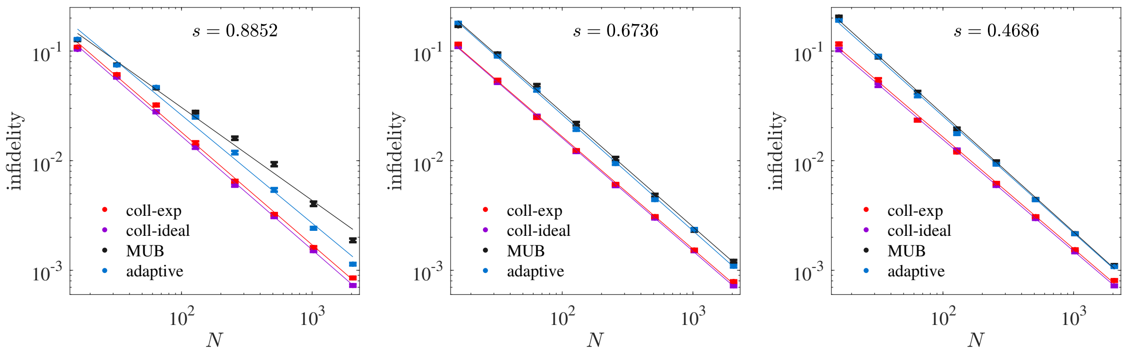

Supplementary Figure 3: Scaling of the mean infidelity in the tomography of mixed states with the collective SIC-POVM (both experiment and simulation). The performances of MUB and two-step adaptive measurements (simulation) are shown for comparison. The three plots correspond to the tomography of three mixed states whose Bloch vectors align with the same direction $\hat{\mathbf{s}} = (0.490, -0.631, 0.602)$ and have lengths $s = 0.885$, 0.674 and 0.469, respectively. Here N is the sample size, ranging from 16 to 2048. Each data point is the average of 1000 repetitions, and the error bar denotes the standard deviation.

A. Scaling of the mean infidelity for mixed states

Three mixed states were considered; their Bloch vectors align with the same randomly-chosen direction $\hat{\mathbf{s}} = (0.490, -0.631, 0.602)$, with lengths $s = 0.885$, 0.674 and 0.469, respectively. The experimental result as well as the simulation result on the ideal collective SIC-POVM are shown in Supplementary Figure **3**. Also shown as benchmarks are the simulation results on the performances of two popular alternative schemes based on mutually unbiased bases (MUB) for a qubit [7–10]. Similar to the case of pure states, the efficiency of the collective SIC-POVM is almost independent of the input state; the infidelity approximately scales as $O(1/N)$ for all states investigated.

By contrast, the scaling behavior for MUB is sensitive to the purity of the input state. When the input mixed state has a high purity (see the left plot in Supplementary Figure **3**), the infidelity scales as $O(1/\sqrt{N})$ when N is small, while it scales as $O(1/N)$ when N is large. The transition region depends on the purity of the input state. In the special case of a pure state ($s = 1$), the $O(1/\sqrt{N})$ scaling approximately holds for all N (see the middle and right plots in Figure 4 in the main text). When the state is highly mixed, MUB achieves almost the same scaling $O(1/N)$ as the collective SIC-POVM, but the infidelity is still larger by a constant factor of about 1.5 (see the right plot in Supplementary Figure **3**).

B. Scaling exponents

In the main text, we investigated the scaling of the mean infidelity $1-F$ achieved by the collective SIC-POVM with the sample size N. Three pure states with Bloch vectors $(0,0,1)$, $\frac{1}{\sqrt{2}}(1,0,1)$ and $\frac{1}{\sqrt{3}}(1,1,1)$ were considered. The experimental result as well as the simulation result on the ideal collective SIC-POVM are shown in Figure 4 in the main text. Also shown as benchmarks are the simulation results on the performances of two popular alternative schemes: one based on mutually unbiased bases (MUB) for a qubit [7–10] and the other based on two-step adaptive measurements proposed in Ref. [11] (cf. Refs. [12–14]). In addition, three mixed states were investigated in Sec. A in this supplement; see Supplementary Figure **3**.

To quantify the distinction between different measurement schemes, experimental data and numerical data are fitted to power laws of the form $1 - F = \beta N^{-p}$. The exponents p are shown in Supplementary Table **2**. According to this table, the efficiency of the collective SIC-POVM is almost independent of the input state; the infidelity approximately scales as $O(1/N)$ for all states investigated. By contrast, the scaling behavior for MUB is very sensitive to the input state. For states with high purities, the scaling is usually much worse except when the input state aligns with one of the POVM elements, which corresponds to "known state tomography" [11].

Supplementary Table 2: Scaling exponents of the mean infidelity $1-F$ against the sample size N in quantum state tomography. The performance of the collective SIC-POVM (both experiment and simulation) is compared with that of MUB and local adaptive measurements (simulation). The first three columns represent the results on three pure states with Bloch vectors as specified, while the last three columns represent results on three mixed states whose Bloch vectors align with the same direction $\hat{\mathbf{s}} = (0.490, -0.631, 0.602)$, with lengths s as specified. The data presented in Figure 4 in the main text and Supplementary Figure **3** are fitted to the formula $1-F=\beta N^{-p}$. The scaling exponents p for the above six states and four measurement schemes are shown in this table. The values inside the parentheses represent the uncertainties in the last two digits of the best-fitted values within 95% confidence intervals.

States	$(0,0,1)$	$\frac{1}{\sqrt{2}}(1,0,1)$	$\frac{1}{\sqrt{3}}(1,1,1)$	$s=0.885$	$s=0.674$	$s=0.469$
coll-exp	1.047(53)	1.002(09)	1.004(12)	1.028(40)	1.025(25)	1.019(37)
coll-ideal	0.999(20)	0.977(20)	1.004(16)	1.036(28)	1.031(14)	1.016(15)
MUB	1.008(11)	0.583(29)	0.571(29)	0.849(86)	1.045(36)	1.074(27)
adaptive	0.970(28)	0.850(54)	0.873(57)	0.986(90)	1.058(25)	1.066(33)

Supplementary Note 5: Implementation of the maximum likelihood estimation

Consider quantum state tomography with a POVM $\{E_j\}_{j=1}^K$ composed of K outcomes. If the quantum system is characterized by the state ϱ, then the probability of obtaining outcome j is $p_j = \mathrm{tr}(\varrho E_j)$. Suppose the POVM is performed N times on N identically prepared quantum systems, and outcome j occurs n_j times with $\sum_j n_j = N$. Now our task is to infer the state of the quantum system from the measurement data $D = \{n_1, n_2, \ldots, n_K\}$.

As a popular estimation strategy in quantum tomography, the ML estimation [15] searches for the quantum state $\hat{\varrho}_{\mathrm{ML}}$ that maximizes the likelihood function, i.e.,

$$\hat{\varrho}_{\mathrm{ML}} := \arg\max_{\varrho} L(D|\varrho), \quad \text{with } L(D|\varrho) = \prod_j p_j^{n_j}. \tag{12}$$

In practice, it is more convenient to work with the normalized log-likelihood function defined as $\mathcal{F}(\varrho) := \frac{1}{N}\ln L(D|\varrho)$. In quantum state tomography with individual measurements, the function $\mathcal{F}(\varrho)$ is concave in ϱ and thus has a unique maximum in the quantum state space, which is convex. In addition, iterative algorithms can be employed by following the gradient

$$G(\varrho) = \sum_j \frac{f_j}{p_j} E_j \tag{13}$$

of $\mathcal{F}(\varrho)$, where $f_j = n_j/N$ is the relative frequency. In quantum state tomography with two-copy collective measurements as considered in this work, however, two-copy quantum states comprise only a subset of the two-qubit state space. Therefore, standard ML algorithms do not apply directly.

Recently, a new optimization strategy, i.e., the accelerated projected-gradient (APG) method was introduced in quantum tomography [16], using which all constraints can be cast into a projection operation. In the current scenario, we have to make sure that the update for ϱ at each iterative step takes on the form $\varrho = \rho^{\otimes 2}$. To this end, we introduce the projection operation $\mathcal{P}$ as follows,

$$\tilde{\varrho} = \mathcal{P}(\varrho): \ \tilde{\varrho} = \tilde{\rho}^{\otimes 2} \text{ with } \tilde{\rho} := \arg\min_{\rho} ||\varrho - \rho^{\otimes 2}||_{\mathrm{HS}}, \tag{14}$$

where $||\cdot||_{\mathrm{HS}}$ denotes the Hilbert-Schmidt norm. This optimization can be done easily by properly parametrizing single-qubit states. Then, we modify the APG algorithm presented in Ref. [16] as follows:

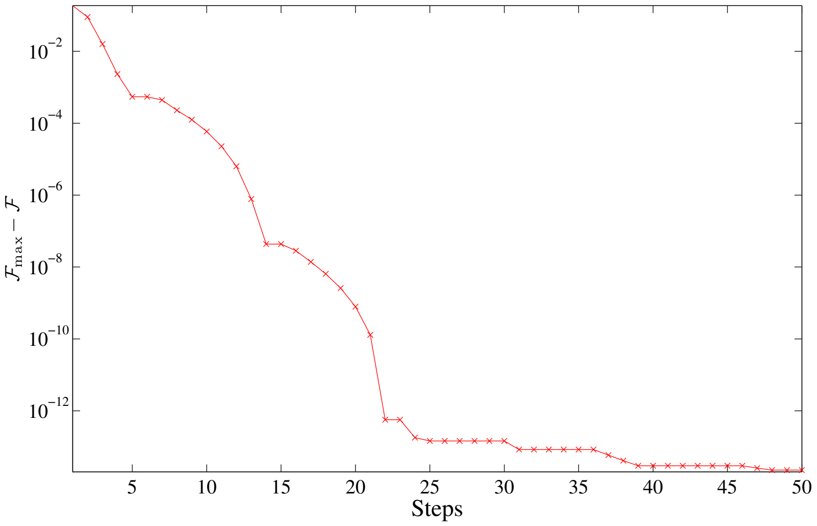

Supplementary Figure 4: Convergence of the APG algorithm in quantum sate tomography with the collective SIC-POVM. The vertical axis represents the deviation $\mathcal{F}_{\max}-\mathcal{F}$ of the normalized log-likelihood $\mathcal{F}$ at each iterative step from the maximum value $\mathcal{F}_{\max}$. In the numerical simulation, the frequencies of obtaining the five outcomes are set to the corresponding probabilities when the collective SIC-POVM is performed on a two-copy state. The APG algorithm is run until the machine precision is reached. The figure shows that the algorithm converges very quickly.

Algorithm: APG for collective measurements

Given $\epsilon > 0$ and $0 < \beta < 1$.
Initialize with any state $\varrho_0 = \rho_0^{\otimes 2}$, $\mathcal{F}_0 = \mathcal{F}(\varrho_0)$; set $\tau_0 = \varrho_0$, $\theta_0 = 1$.
for $k = 1, 2, \cdots$, **do**
  Update $\varrho_k = \mathcal{P}[\tau_{k-1} + \epsilon\, G(\tau_{k-1})]$ according to Eq. (14), $\mathcal{F}_k = \mathcal{F}(\varrho_k)$.
  Termination criterion!
  if $\mathcal{F}_k < \mathcal{F}_{k-1}$ **then** (Restart)
    Reset $\epsilon = \beta\epsilon$, $\varrho_k = \varrho_{k-1}$, $\tau_k = \varrho_k$, and $\theta_k = 1$.
  else (Accelerate)
    Set $\theta_k = \frac{1}{2}\left(1 + \sqrt{1 + 4\theta_{k-1}^2}\right)$, then update $\tau_k = \varrho_k + \frac{\theta_{k-1}-1}{\theta_k}(\varrho_k - \varrho_{k-1})$.
  end if
end for

Generally speaking, the APG algorithm works in a similar way to those conventional gradient approaches, but with a tweaked gradient direction in each step to boost the convergence. Specifically, each update of the target ϱ in APG is based on another state τ, which gives each update some "momentum" from the previous step. The momentum is controlled by the parameter θ, which is reset to 1 whenever it causes the current step to point too far from the direction specified by $G(\cdot)$. Upon convergence, ϱ and τ will eventually merge to the same point. For more technical details about the APG algorithm, e.g., the 'Restart' and 'Accelerate' operations, see Ref. [16] and references therein.

Supplementary Figure 4 illustrates the convergence of the APG algorithm applied to the collective SIC-POVM. In the numerical simulation, a qubit state ρ is generated randomly, and the frequency of obtaining each outcome is set to the corresponding probability, that is, $f_j = p_j = \mathrm{tr}(\rho^{\otimes 2} E_j)$, where E_j for $j = 1$ to 5 are the five outcomes of the collective SIC-POVM. In this example, the maximum value $\mathcal{F}_{\max}$ of the normalized log-likelihood function is attained at the true state ρ; the deviation $\mathcal{F}_{\max} - \mathcal{F}$ from the maximum value is plotted as a function of the number of steps. The figure shows that the APG algorithm converges very quickly.

Supplementary References

[1] Kurzyński, P. & Wójcik, A. Quantum walk as a generalized measuring device. *Phys. Rev. Lett.* **110**, 200404 (2013).

[2] Bian, Z. *et al.* Realization of single-qubit positive-operator-valued measurement via a one-dimensional photonic quantum walk. *Phys. Rev. Lett.* **114**, 203602 (2015).

[3] Zhao, Y.-Y. *et al.* Experimental realization of generalized qubit measurements based on quantum walks. *Phys. Rev. A* **91**, 042101 (2015).

[4] Zauner, G. Quantum designs: Foundations of a noncommutative design theory. *Int. J. Quantum Inform.* **09**, 445–507 (2011).

[5] Renes, J. M., Blume-Kohout, R., Scott, A. J. & Caves, C. M. Symmetric informationally complete quantum measurements. *J. Math. Phys.* **45**, 2171 (2004).

[6] Fiurášek, J. Maximum-likelihood estimation of quantum measurement. *Phys. Rev. A* **64**, 024102 (2001).

[7] Wootters, W. K. & Fields, B. D. Optimal state–determination by mutually unbiased measurements. *Ann. Phys.* **191**, 363 (1989).

[8] Durt, T., Englert, B.-G., Bengtsson, I. & Życzkowski, K. On mutually unbiased bases. *Int. J. Quantum Inform.* **08**, 535 (2010).

[9] Adamson, R. B. A. & Steinberg, A. M. Improving quantum state estimation with mutually unbiased bases. *Phys. Rev. Lett.* **105**, 030406 (2010).

[10] Zhu, H. Quantum state estimation with informationally overcomplete measurements. *Phys. Rev. A* **90**, 012115 (2014).

[11] Mahler, D. H. *et al.* Adaptive quantum state tomography improves accuracy quadratically. *Phys. Rev. Lett.* **111**, 183601 (2013).

[12] Kravtsov, K. S. *et al.* Experimental adaptive Bayesian tomography. *Phys. Rev. A* **87**, 062122 (2013).

[13] Hou, Z., Zhu, H., Xiang, G.-Y., Li, C.-F. & Guo, G.-C. Achieving quantum precision limit in adaptive qubit state tomography. *npj Quantum Information* **2**, 16001 (2016).

[14] Qi, B. *et al.* Adaptive quantum state tomography via linear regression estimation: Theory and two-qubit experiment. *npj Quantum Information* **3**, 19 (2017).

[15] Paris, M. G. A. & Řeháček, J. (eds.) *Quantum State Estimation*, vol. 649 of *Lecture Notes in Physics* (Springer, Berlin, 2004).

[16] Shang, J., Zhang, Z. & Ng, H. K. Superfast maximum-likelihood reconstruction for quantum tomography. *Phys. Rev. A* **95**, 062336 (2017).
